# Impact of the K-line in patients with ossification of the posterior longitudinal ligament: Analysis of sagittal cervical curvature changes and surgical outcomes

**DOI:** 10.3389/fsurg.2023.1095391

**Published:** 2023-02-15

**Authors:** Zhongxin Tang, Tailong Chen, Jun Tan, Huafeng Zhang

**Affiliations:** Department of Orthopaedic Surgery, The First Affiliated Hospital of Zhengzhou University, Zhengzhou, China

**Keywords:** cervical, OPLL, surgical outcomes, laminoplasty, K-line

## Abstract

**Objective:**

This study aimed to investigate the relationship of the K-line with sagittal cervical curvature changes and surgical outcomes in patients with cervical ossification of the posterior longitudinal ligament (OPLL).

**Methods:**

We retrospectively reviewed 84 patients with OPLL who underwent posterior cervical single-door laminoplasty. The patients were divided into a K-line-positive (+) group and a K-line-negative (−) group. Perioperative data, radiographic parameters, and clinical outcomes were compared between the two groups.

**Results:**

Of 84 total patients, 50 patients were in the K (+) group and 29 patients were in the K (–) group. Neurological function improved in both groups after laminoplasty. The C2–7 Cobb angle, T1 slope, and C2–7 sagittal vertical axis were significantly changed in the K(−) group compared with those in the K (+) group before the operation and at the 3-month and final follow-ups.

**Conclusion:**

Neurological function was recovered in both groups, and the clinical effect on the K (+) group was better than that on the K (−) group. The cervical curvature in patients with OPLL tends to be anteverted and kyphotic after laminoplasty and is an important factor in reducing the clinical effect.

## Introduction

Ossification of the posterior longitudinal ligament (OPLL) is one of the main causes of cervical myelopathy (1), which not only causes the spinal cord and nerve root disease in patients but also increases the risk of spinal cord injury after minor trauma (2, 3). Its pathogenesis is unclear, as many factors are at play. These include endocrine factors (4, 5), genetic factors (6, 7), mechanical stress stimuli, and biomechanical factors (8). Continued ossification often results in cervical spinal stenosis and progressive compression of the nervous system, so patients with OPLL often require surgical treatment. Laminoplasty is a commonly used surgical method. However, many factors may lead to poor symptom relief or even aggravation after posterior laminoplasty (9).

Fujiyoshi et al. (10) proposed the K-line theory to make an appropriate prognosis evaluation for OPLL patients. The K-line is a virtual line that connects the midpoints of the anteroposterior diameter of the spinal canal at C2 and C7 in a plain lateral radiogram. If the peak of the OPLL ossification focus does not exceed this line, it is K-line-positive; otherwise, it is K-line-negative. This single parameter can be used to explain the poor surgical outcomes after laminoplasty due to cervical kyphosis and the high occupancy rate of OPLL to the spinal canal. Some researchers (11, 12) have suggested that patients who are K-line-negative usually have poor outcomes after laminoplasty due to limited spinal cord retromobility. However, the K-line classification does not include dynamic factors, and there is controversy regarding whether sagittal cervical curvature will affect the efficacy of laminoplasty in OPLL patients.

Therefore, we designed the present study to (1) analyze the correlation between the clinical efficacy of laminoplasty and the change in cervical curvature in patients with OPLL and (2) analyze how the K-line is related to changes in the sagittal cervical curvature and kyphosis after laminoplasty.

## Materials and methods

### Patients

The study design was approved by the ethics committee of our institution. We retrospectively reviewed the medical records of patients who underwent posterior cervical single-door laminoplasty for cervical myelopathy caused by OPLL at our institution between January 2015 and December 2019. Altogether, 84 patients were ultimately included in this study. The inclusion criteria are as follows: (1) diagnosis of cervical compressive myelopathy due to OPLL; (2) increased signal intensity of the spinal cord on MRI; and (3) OPLL involving two or more vertebrae. The exclusion criteria are as follows: (1) cervical trauma and tumor; (2) a history of cervical surgery; (3) a history of neuromuscular diseases or the presence of other complex concomitant diseases; and (4) follow-up of less than 2 years.

### Operative procedure

For each patient, we performed single-open-door laminoplasty. A midline incision was made on the posterior neck skin to expose the laminae and articular processes of the decompression segment. Determined by the severity of the symptoms, the more severe side of the laminae was selected as the open-door side, with the other side used as the hinged side. The grooves along the junction of the lamina and facet articular process on both sides were cut by a high-speed grinding drill or an ultrasonic bone knife. After the laminae had been elevated, anchor sutures were used and ﬁxed. After postoperative day 1, the patients were permitted to ambulate using their neck bracket.

### Data collection

**Figure 1:**
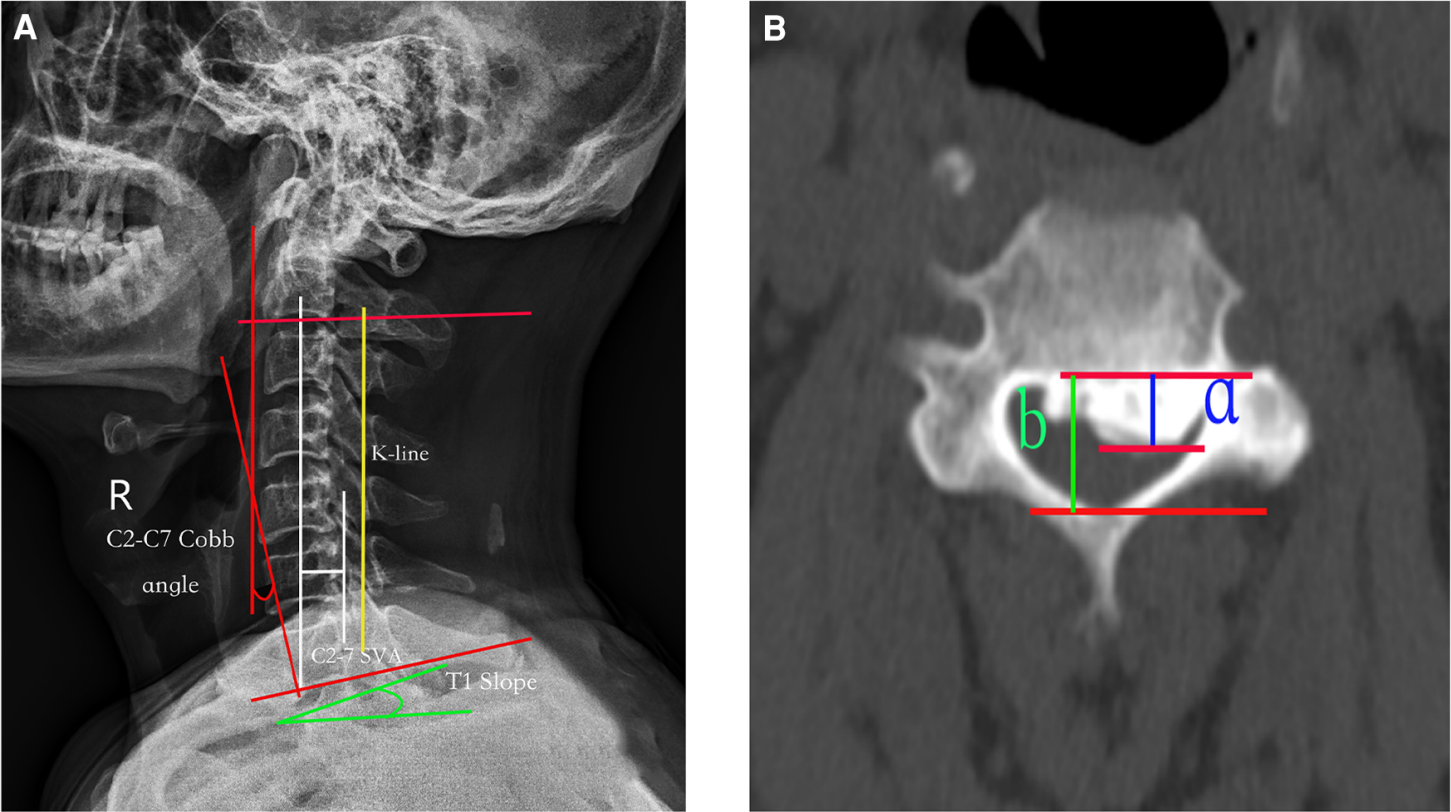
(**A**) Radiographic measurements: (1) the K-line is a straight line joining the midpoints of the spinal canal at C2 and C7 on a lateral radiograph; (2) C2–C7 SVA: the distance between the C2 plumb line and the superoposterior endplate of C7; (3) C2–C7 Cobb angle: the angle formed by the C2 and C7 lower endplates; and (4) T1 slope: the angle between the horizontal line and the T1 upper endplate. (**B**) Percentage of spinal canal occupation by ossified mass. The thickness of the ossification block (a, green line) and the anteroposterior diameter of the spinal canal (b, yellow line) were measured on the axial CT section of the highest point of the ossification block. The percentage of spinal canal occupation by the ossification block = a/b × 100%. SVA, sagittal vertical axis.

The mean follow-up period was 36 months (ranging from 24 to 84 months). All patients were re-examined 3 months after the operation and at the last follow-up appointment. Based on the K-line (10) (Figure 1), the patients were retrospectively classified into the K-line-positive [K (+)] group and the K-line-negative [K (−)] group. The basic data of each patient included age, sex, and body mass index (BMI). The sagittal cervical radiographic measures included x-ray radiography, CT, and MRI. The C2–7 sagittal vertical axis (SVA), C2–7 Cobb angle, T1 slope, and spinal canal occupation rate of the ossified mass were measured (Figure 1). The Japanese Orthopedic Association (JOA) score (17-point method) (13) was used to evaluate the clinical outcomes before the operation, 3 months after the operation, and at the last follow-up. The improvement rate of the JOA score 3 months after the operation and at the last follow-up was also calculated. Recovery rate (%) = (postoperative JOA score – preoperative JOA score/17 – preoperative JOA score) × 100.

### Statistical analysis

SPSS 22.0 statistical software (IBM Corp, Armonk, New York, United States) was used for the statistical analysis of the data. The data are expressed as the mean ± SD. The analyses of the continuous variables were performed using unpaired Student's *t*-test or Welch's test for two-group comparisons, and the clinical and radiological measurements were analyzed using Wilcoxon’s test. Differences with a *P*-value of <0.05 were considered statistically significant.

## Results

A total of 84 patients were reviewed, and the duration of follow-up ranged from 24 to 84 months. There were 51 men and 33 women, with an average age of 55.60 ± 8.94 years (range 35–7 76 years). According to the position in relation to the K-line, 59 patients were in the K (+) group (36 men, 23 women) and 25 patients were in the K (−) group (15 men, 10 women). There were no significant differences in the characteristics of the data between the groups (*P* > 0.05; Table 1).

**Table 1:** Comparison of patient characteristics between the groups.

Variable	K (+) group (59 patients)	K (–) group (25 patients)	*P*-value
Age (years)	55.78 ± 9.41	55.15 ± 7.89	0.769
Gender (female/male; numbers)	23/36	10/15	0.559
BMI (kg/m^2^)	25.48 ± 3.87	23.87 ± 1.88	0.052
Canal occup. ratio	46.60 ± 12.11	54.16 ± 10.44	0.662

**Figure 2:**
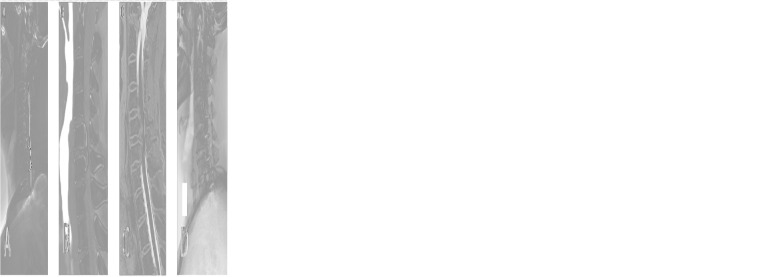
(**A**) A 60-year-old woman with OPLL and negative K-line. (**A**) A preoperative x-ray film of the cervical spine showing that the peak of ossification foci is beyond the K-line; (**B**) preoperative sagittal CT showing that the range of ossification of the posterior longitudinal ligament is C5–C6; (**C**) preoperative MRI showing obvious compression in front of the cervical spinal cord; and (**D**) postoperative x-ray showing good internal fixation position and lordosis reduced.

There were significant differences in C2–7 SVA, C2–7 Cobb angle, and T1 slope between the two groups before the operation (*P* < 0.001). In the K (+) group, the T1 slope and the Cobb angle were both larger than those in the K (−) group, and the C2–7 SVA was smaller than that in the K (−) group. After the operation, the T1 slope and the Cobb angle decreased in both groups, and the change was greater in the K (−) group (*P* < 0.05). However, the C2–7 SVA increased more in the K (+) group than in the K (−) group (*P* < 0.05). Meanwhile, there were no significant differences in the JOA scores between the groups before and after the operation (*P* > 0.05), and there were significant differences in the JOA improvement rate (*P* < 0.05; Table 2 and Figure 2).

**Table 2:** Comparison of surgical results between the groups.

	K (+) group (59 patients)	K (–) group (25 patients)	*P*-value
**C2–7 Cobb angle (°)**			
Preoperative	18.77 ± 6.70	11.28 ± 6.67	0.010
Postoperative 3 months	17.04 ± 6.77	8.88 ± 6.48	0.003
Postoperative change	1.73 ± 0.68	2.39 ± 0.82	<0.001
At last follow-up	15.59 ± 4.98	7.08 ± 3.80	<0.001
**C2–7 SVA (mm)**			
Preoperative	17.51 ± 5.67	20.51 ± 5.88	0.020
Postoperative 3 months	20.09 ± 6.55	23.72 ± 6.21	<0.001
Postoperative change	2.58 ± 1.88	3.21 ± 1.52	0.010
At last follow-up	21.89 ± 7.61	26.70 ± 7.11	0.050
**T1 slope (°)**			
Preoperative	23.34 ± 5.58	21.58 ± 5.66	0.192
Postoperative 3 months	21.64 ± 5.7	18.94 ± 5.52	0.048
Postoperative change	1.70 ± 0.54	2.64 ± 1.01	<0.001
At last follow-up	19.46 ± 6.86	17.29 ± 5.38	0.436
**JOA score**			
Preoperative	11.41 ± 2.11	9.6 ± 2.16	0.562
Postoperative 3 months	14.43 ± 2.32	12.60 ± 2.27	0.763
Improvement rate of the JOA score (%)	62.499 ± 26.66	44.37 ± 15.22	0.002
At last follow-up	15.21 ± 2.21	13.61 ± 2.16	0.030

SVA, sagittal vertical axis; JOA, Japanese Orthopedic Association.

## Discussion

OPLL often causes abnormal paranesthesia and motor dysfunction due to spinal stenosis with compression of the spinal cord and nerve roots. There are commonly two surgical approaches for removing cervical OPLL: anterior and posterior. The anterior approach is riskier and more prone to spinal cord injury (14, 15), so posterior spinal cord decompression is widely performed to treat patients with OPLL (12, 16, 17). Single-open-door laminoplasty can achieve decompression and preserve spine stability to a certain extent. It has yielded favorable clinical outcomes when used for treating OPLL (18–20). However, there are many disadvantages of posterior laminoplasty (11): decompression is achieved through spinal cord retreat to the dorsal side rather than direct decompression. As the dentate ligament connects the spinal cord to the front of the spinal canal, and the nerve roots from the dura and the front of the spinal cord also limit the movement of the spinal cord to the back. Therefore, if the spinal cord does not move backward enough, the compression of the ossification focus in front of the spinal cord will persist, and the postoperative outcomes will be poor.

Iwasaki et al. (21) concluded that laminoplasty is effective and safe for most patients with an occupying ratio of OPLL of less than 60% but is poor or fair in patients with an occupying ratio greater than 60%. Koda et al. (22) found that laminoplasty should not be used for K-line (–) cervical OPLL. In the present study, the JOA improvement rate was significantly better in the K (+) group than in the K (−) group at the last follow-up (*P* < 0.05). This shows that the efficacy of laminoplasty in the K (+) group is significantly better than that in the K (–) group. The possible reason is that the spinal cord did not give way to the back after laminoplasty in the K (−) group, and the improvement in neurological symptoms was not noticeable. Therefore, it is inappropriate for such patients to choose posterior laminoplasty, and anterior surgery should be the first choice.

The destruction of cervical muscles and ligaments after posterior laminoplasty may lead to changes in cervical curvature, which accelerates the change in cervical curvature, leading to increased cervical anteversion and reduced cervical lordosis. In patients with cervical spondylosis, the center of gravity of the cervical vertebra must be moved backward to achieve sagittal balance. In our research, the patients in the K (−) group of the present study showed a significant change in cervical spine curvature after posterior laminoplasty (the T1 slope decreased, the C2–7 Cobb angle decreased, and the C2–7 SVA increased). Miyazaki et al. (23) and Cho et al. (24) concluded that their clinical outcomes demonstrated overall improvement after cervical laminoplasty with cervical OPLL, regardless of the preoperative T1 slope. Kim et al. (25) showed that preoperative cervical lordosis is not related to the clinical effect after laminoplasty. In contrast, Suk et al. (26) found that preoperative lordosis of the cervical spine is a prerequisite for laminoplasty, and maintaining postoperative lordosis is also important for decompression of the spinal cord. Masaki et al. (11) stated that the sagittal position of the cervical spine often showed kyphosis after laminoplasty but that cervical sagittal alignment and clinical outcomes were still unclear.

We found that the clinical effect was connected to the change in sagittal curvature of the cervical spine. Compared to the K (+) group, there were significant changes in the cervical spine curvature in the K (–) group, and the clinical effect was also poor at the last follow-up (*P* < 0.05). We also found that the change in the cervical curvature after posterior laminoplasty tended toward anteversion and kyphosis in both groups. However, the preoperative cervical lordosis of the patients in the K (+) group was greater than that in the K (–) group, thus buffering the changes in cervical lordosis after surgery. Based on the bowstring effect, the K (+) group had a better clinical effect after surgery. In contrast, for patients in the K (–) group, the preoperative cervical curvature was not sufficient to resist the postoperative curvature change, and patients were more affected by the cervical curvature, leading to a worse clinical effect. Therefore, we believe that the change in cervical curvature after laminoplasty may influence the clinical effect in patients with OPLL.

In the present study, we still have several limitations. (1) There were a limited number of eligible patients, as this was a single-center study. (2) We only included patients who underwent single-open-door laminoplasty. Larger studies with long-term follow-ups are needed.

## Conclusion

Neurological function was improved after posterior laminoplasty in both K-line groups. The clinical effect in the K (+) group was better than that in the K (−) group. In addition, the cervical curvature changed little in the K (+) group compared with the K (–) group. The important factor reducing the clinical effect of laminoplasty is that cervical curvature in patients with OPLL tends toward anteversion and kyphosis.

## Data Availability

The raw data supporting the conclusions of this article will be made available by the authors, without undue reservation.
